# A Comprehensive Approach to Powder Feedstock Characterization for Powder Bed Fusion Additive Manufacturing: A Case Study on AlSi7Mg

**DOI:** 10.3390/ma11122386

**Published:** 2018-11-27

**Authors:** Jose Alberto Muñiz-Lerma, Amy Nommeots-Nomm, Kristian Edmund Waters, Mathieu Brochu

**Affiliations:** 1 REGAL Aluminum Research Center, Department of Mining and Materials Engineering, McGill University, Montreal, QC H3A 0C5, Canada; jose.muniz@mcgill.ca (J.A.M.-L.); amy.nomm@mcgill.ca (A.N.-N.); 2 Department of Mining and Materials Engineering, McGill University, Montreal, QC H3A 0C5, Canada; kristian.waters@mcgill.ca

**Keywords:** additive manufacturing, metal powders, powder flowability, powder properties, aluminum, water absorption

## Abstract

In powder bed fusion additive manufacturing, the powder feedstock quality is of paramount importance; as the process relies on thin layers of powder being spread and selectively melted to manufacture 3D metallic components. Conventional powder quality assessments for additive manufacturing are limited to particle morphology, particle size distribution, apparent density and flowability. However, recent studies are highlighting that these techniques may not be the most appropriate. The problem is exacerbated when studying aluminium powders as their complex cohesive behaviors dictate their flowability. The current study compares the properties of three different AlSi7Mg powders, and aims to obtain insights about the minimum required properties for acceptable powder feedstock. In addition to conventional powder characterization assessments, the powder spread density, moisture sorption, surface energy, work of cohesion, and powder rheology, were studied. This work has shown that the presence of fine particles intensifies the pick-up of moisture increasing the total particle surface energy as well as the inter-particle cohesion. This effect hinders powder flow and hence, the spreading of uniform layers needed for optimum printing. When spherical particles larger than 48 µm with a narrow particle distribution are present, the moisture sorption as well as the surface energy and cohesion characteristics are decreased enhancing powder spreadability. This result suggest that by manipulating particle distribution, size and morphology, challenging powder feedstock such as Al, can be optimized for powder bed fusion additive manufacturing.

## 1. Introduction

Laser powder bed fusion (LPBF), an additive manufacturing (AM) process that allows the fabrication of complex geometries, is creating opportunities to fabricate innovative parts with enhanced functionality [1]. Aluminum alloys have shown to be of great interest in the AM sector acting as an economical method to manufacture parts that cannot be produced through traditional casting or forging routes. However, aluminum powders are amongst the most difficult powders to process via LPBF due to the lack of repeatability associated with the quality of powder feedstock [2]. 

The powder feedstock quality can be assessed by its extrinsic and intrinsic properties. Extrinsic properties refer to the relationship between powder morphology, including particle size distribution and shape, to the final part performance. While intrinsic properties refer to the influence of the powder composition and microstructure on the final quality of the additively manufactured part [3]. 

In terms of extrinsic properties, it is well accepted that spherical powder particles positively contribute towards powder flowability and higher particle packing densities in the powder bed [4]. Spherical morphologies are also typically associated with low levels of particle-particle friction which results in enhanced powder flow. Another important extrinsic property is the apparent density of the powder feedstock; some reports in literature have suggested that this factor has the most significant effect on the final density of the manufactured parts [5,6]. Apparent density is directly influenced by a variety of factors such as particle size distribution (PSD), particle shape, inter-particle friction, surface chemistry, and agglomeration. However, among these factors, the particle shape, size and distribution are suggested to be the most influential [7]. Secondarily to this, it has recently been reported that even with powder feedstock optimisation, particle segregation can occur within a powder bed affecting the local apparent density [8,9] and the mechanical properties of the produced part. 

For LPBF, suppliers typically provide powders in the size range of 15–45 µm. However, selecting an appropriate powder size and size distribution is a key factor as even within this range, competitive powder interactions can occur. Theoretically, the presence of fine particles in the powder feedstock (smaller than the recoating layer thickness) would be beneficial. As this would lead to an increase in the powder density which would improve the surface roughness and reduce the number of defects in the printed parts [10,11]. Smaller particle sizes are also known to have increased laser energy absorption, thus improving their processing. The advantages gained from fine particles are contradicted by the significant disadvantages associated with their use, such as their high tendency to agglomerate [12], and their large surface area to mass ratio. The agglomeration of fine powders are more likely to promote pore and void formation during processing due to the irregular shapes and variable sizes of the agglomerates formed [13]. Additionally, agglomerated powders are known to increase the reflectivity of the powder bed resulting in less energy absorption during the manufacturing process [12,14]. The discussed factors makes the spreading of uniform powder layers more challenging thus compromising the process stability since inhomogeneous powder bed densities give rise to non-uniform heating increasing the risk of defect formation [12,13,15].

Intrinsic properties are those which relate to the powder composition and microstructure of the resulting part. Here, issues arise due to powder stability. Metallic powder composition during storage, handling and building (including re-use), can have significant changes over time due to oxygen and moisture exposure. This phenomenon is significantly influenced by the surface-to-volume ratio of the powder. Both oxygen and moisture contamination are known to produce reaction products that can change and degrade the microstructure of the particles, resulting in an increased particle porosity and ultimately reducing the mechanical properties such as ductility and toughness of 3D printed parts [2]. 

Some factors affect both the extrinsic and intrinsic properties. One of these factors is the likelihood of elemental evaporation of alloying elements during the LBPF process. Smaller powder particles are more susceptible to this phenomenon due to their high laser absorptivity [15,16]. As discussed there are a wide number of aspects which need to be considered when looking to optimize suitable powder feedstock for the LPBF process. Reaching the perfect balance in terms of particle size distribution, contamination and printability is not a trivial process.

Recently, a new atomization technique has been developed that produces metallic powders with high sphericity, narrow PSD and uniform microstructure [17]. The powders formed are expected to provide superior properties compared to typical gas atomized powders for AM applications, leading to a higher density and improved mechanical properties in the final part.

The purpose of this paper is to compare the powder properties of various AlSi7Mg powders obtained from different production methods. Different characterization techniques have been employed to obtain information regarding the apparent density, powder flow, spread density, moisture sorption, surface energy and work of cohesion of the studied powders. The conclusions contribute to the growing knowledge on powder characterization methods and optimum powder feedstock properties for AM processing.

## 2. Materials and Methods 

Three powders produced using the gas atomization principle were used in the current study. The powders are termed A, B, and C, and are all based on the A356-A357 systems. Scanning electron microscopy (SEM) was used to characterize the morphology of the powders using a Hitachi SU3500 Scanning Electron Microscope, Tokyo, Japan. The powder PSD was obtained by a Horiba laser particle size analyser (Model LA-920, Kyoto, Japan). The log-normal slope parameter method was used to calculate the dimensionless PSD width (S_w_) which represents the breadth of the PSD [18]. In this method, the standard deviations of the cumulative distribution were plotted against the logarithmic particle size resulting in a linear plot. The slope of the linear plot represents the S_w_ while the intersection with the x-axis is equivalent to the median particle diameter, D_50_. If the particle size is very narrow, high values of S_w_ are expected.

Powder flowability was measured using Hall and Carney funnels (QPI-HFM1800SS, Qualtech Products Industry, Denver, CO, USA) according to the ASTM standards [19,20]. Apparent density was assessed using the Hall and Carney funnels, as well as the Arnold meter according to the ASTM standards B212, B417, and B703, respectively [21,22,23]. The latter method, not commonly studied for AM characterization, is representative of a gravity fed powder delivery mechanism. Theoretical densities used for the calculation of the apparent density were 2.70 g/cm^3^ for powders A and B and 2.68 g/cm^3^ for powder C.

Powder spread density was studied using a single layer of powder with a thickness of ~100 µm CT scanned by a Zeiss Xradia 520 Versa 3D X-ray microscope, Oberkochen, Germany. A bespoke testing holder made of stainless steel 316L was printed using a Renishaw AM250 (Gloucestershire, UK) to simulate the build plate. The testing holder was then stuck onto a cylindrical aluminum mounting rod. Once the testing holder was fixed in the CT holder, the powder from the batch of interest was spread over the simulated built plate region using the doctor blade technique and covered by a centrifuge tube to limit movement of the powders during testing. Each tomography scan was collected using the HE2 filter with sample rotation of 360°, voltage of 60 KV, current of 82 µA, and an exposure time of 18 s. A magnification of 4× objective was selected to provide a 3D isotropic voxel size of 1 µm across the scanned volume. After scanning, the acquired micro-computed tomography images were segmented using the open source image-processing platform imageJ Fiji (ImageJ 1.52b, National Institute of Health, Bethesda, MD, USA) [24] and reconstructed using the Dragonfly software version 3.1.0.319 from Object Research Systems (ORS, Montreal, QC, Canada) [25]. Finally, the total particle volume as a percentage occupied within the scanned area was considered as the powder spread density.

In order to study the particle segregation within a single 50 µm thick layer, the surface of an Al built plate was patterned by rastering the laser of a Renishaw AM400 to simulate the surface roughness created in a part during printing. The powder was then spread over the Al plate using the Renishaw AM400 spreading system, Gloucestershire, UK). Once the powder layer was deposited over the plate, representative samples were collected from the top and bottom sections for PSD analysis by SEM to determine if powder segregation occurred.

The moisture sorption behavior of the studied powders was evaluated using the gravimetric dynamic vapor sorption (DVS) technique (DVS Intrinsic Plus, from Surface Measurement Systems, London, UK). The DVS Intrinsic apparatus measures mass change (±0.1 µg) under controlled temperature and humidity. Prior to testing, samples were individually dried under vacuum at 200°C. Dried powder samples (~100 mg) were loaded into an aluminum pan and placed into a chamber kept at 25 °C, and allowed to reach equilibrium, i.e., until the change in mass as a function of time was less than 0.002% per minute. Testing started by reaching 0% relative humidity (RH) and soaked for 12 h. Then, the RH was increased in steps of 10% until 80% RH was reached. Each RH step was held until equilibrium. Once 80% RH had been reached, the RH was ramped down to 0% in steps of 10 mirroring the ramp up.

To evaluate the effect of humidity on the flowability of the tested powders, a multiple cycle flowability test using the Carney funnel was performed. The test consisted on drying 50 g of each powder at 200 °C under vacuum for a period of 2 h. Immediately after drying, Carney flow tests were carried out continuously using the same powder at a 40% RH environment until a total of 30 measurements were obtained.

The surface energy, defined as the energy per unit area required to create a new interface and described as the sum of the dispersive component (London dispersion forces) and the specific component (dipole-dipole, induced dipole, and hydrogen bonding interactions), was determined using inverse gas chromatography (IGC) [26,27]. Details of the method can be found in ref. [28]. Pre-salinized glass columns (300 mm length, 4 mm inner diameter) were filled with the different powders (A, B, and C), and the ends plugged with salinized glass wool. The columns were then placed in a surface energy analyser (IGC-SEA, from Surface Measurement Systems, London, UK), with helium flowing through at 10 mL·min^−1^ as an inert carrier gas. Methane was used for dead volume corrections. All measurements were carried out at 30 °C and 0% RH. In order to determine the specific surface area of the samples, octane was passed through each column at set molecular amounts, and an isotherm was produced. From this isotherm, the BET equations were used to determine the specific surface area. To determine the dispersive component of the surface energy, alkane probes with increasing chain length from heptane to nonane (HPLC grade, Sigma Aldrich, St. Louis, MO, USA), were passed through the sample at a set (fractional) surface coverages from 0.1 to 0.3. Sufficient time was allowed between injections as to allow for the probe to pass completely through the column. For the specific surface energy component, dichloromethane and toluene (both HPLC grade, Sigma Aldrich, St. Louis, MO, USA) were used as the polar probes.

Two different techniques, static and dynamic angles of repose, were also used to assess the powders cohesiveness. The static angle of repose consists on freely flowing powders through a funnel to form a characteristic conical heap onto a horizontal plate. The angle developed between the surface of the conical heap and the plate represents the static angle of repose. For this purpose, 50 g of as-received powders were dispensed through a Carney funnel onto the center of a plate. The angle of repose was then measured between the surface of the powder heap and the plate. This procedure was repeated three times for each powder and the average values are reported.

The dynamic angle of repose measurement was obtained using the granular material flow analyzer, Granudrum® (Granutools™, Awans, Belgium). The analysis consists of a transparent drum that is filled with 50 cm^3^ of powder and rotated at an angular velocity to induce powder flow. The rotating drum is backlight and a camera is used to capture images of the avalanche at different times. Angular velocities ranging from 2–20 rpm were used. For each angular velocity, 50 images of the drum separated by 1000 ms were acquired. The interface location between the air and powder was automatically detected and the average position as well as the deviations around this average position were automatically computed for each velocity. The dynamic angle of repose was determined from the center of the flow and the deviations from the interface was directly related to the cohesion inside the drum and denominated as the cohesion index [29]. The process was repeated three times and the average values are presented.

## 3. Results

### 3.1. Powder Morphology and Particle Size Distribution

**Figure 1:**
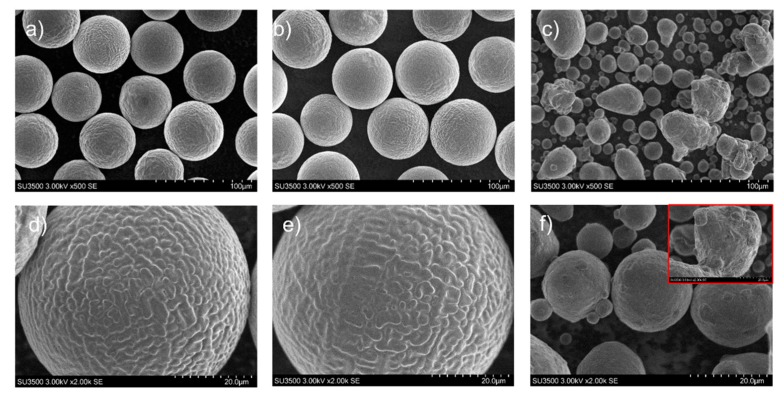
Representative micrographs of the powders tested within this study: (**a**) powder A, (**b**) powder B, (**c**) powder C, and corresponding high magnification images of (**d**) powder A, (**e**) powder B, (**f**) small particles of powder C. The red square in image (**f**), corresponds to the high magnification image of large particles encountered on image (**c**).

The powder morphology corresponding to powders A, B, C imaged by SEM are shown in Figure 1a–f. Spherical particles with regular morphologies, smooth surfaces, and a limited number of satellites were observed for powders A and B. In comparison, the particles present in powder C were more varied in their nature with irregular surfaces; the smaller particles were spherical, but the larger particles were distorted with satellites present. 

**Figure 2:**
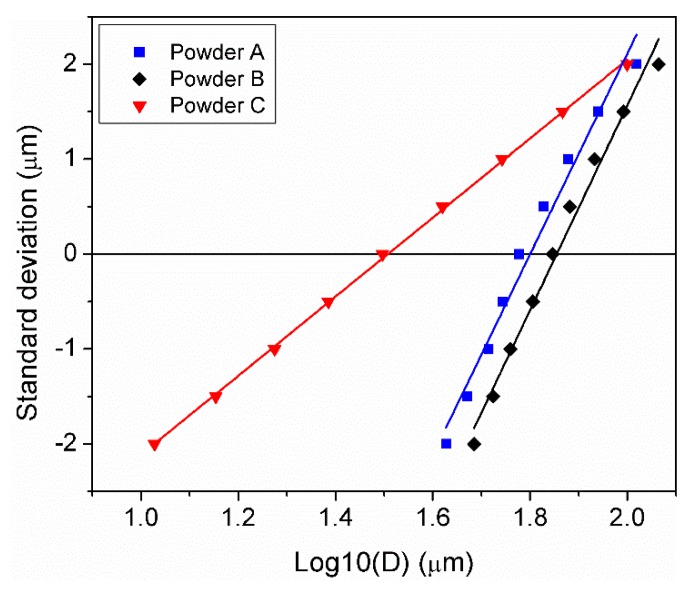
Graphical representation of the cumulative logarithmic particle size versus the standard deviation of powders A, B, and C.

The PSD expressed in terms of the “log-normal slope” parameter method is a way to easily compare and identify in a single plot the S_w_ and the median value of the PSD. A graphical representation of the cumulative particle size against the standard deviations used to determine the S_w_ of powders A, B, and C is depicted in Figure 2. It was clearly seen that powder C presents the lowest gradient with finer modal particles when it was compared with powders A and B. The corresponding PSD D_10_, D_50_, D_90_, and the dimensionless S_w_ values are summarised in Table 1. Powders A and B present a narrow PSD distribution with S_w_ values of 10.6 and 10.8 respectively, while powder C shows a S_w_ of 4.2 indicating a wide PSD.

### 3.2. Density and Flow Characteristics 

Apparent density and flow characteristics are reported to provide useful insight into how the powders will pack in a loose state, which simulates the powder layer spreading during AM processing where no compression or tapping forces are applied [30]. Table 2 presents the flow characteristics of powders A, B, and C, obtained by the Hall and Carney funnels. The measured flow times under these characterization methods indicate no statistical difference between powders A and B. In contrast, powder C showed a lack of flow through the Hall funnel while for the Carney funnel, the measured flow corresponds to 15.3 ± 0.4 s for 50 g of powder. The results obtained by both techniques, indicate that powders A and B exhibit better flow behaviors than powder C. 

Table 3 summarizes the apparent density values obtained by three different traditional techniques recommended for powder metallurgy, i.e., Hall funnel, Carney funnel, and Arnold meter. Powder A presents the highest apparent density followed by powder B and powder C. It is known that the hall funnel is unable to quantify the apparent density of non-flowing powders. However, alternative techniques used in the powder metallurgy field such as the Carney funnel and Arnold meter are able to provide consistent values with a difference of approximately 1% between each technique.

### 3.3. Spread Density

**Figure 3:**
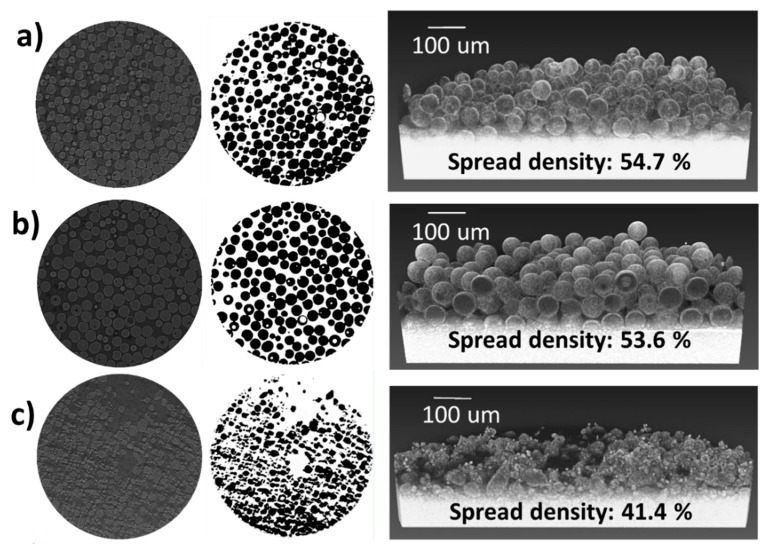
2D and 3D reconstructed images of (**a**) powder A, (**b**) powder B, and (**c**) powder C used to determine the spread density.

The laser-powder interactions during LPBF processing influence the melt pool dynamics. Ideally, during powder spreading, a homogeneous powder bed with high packing density would be preferred. In order to evaluate the spread density, X-ray micro-computed tomography. This technique provides a 3D visualization of particles, which allows the determination of the internal porosity as well as the packing density of powders contained in a powder bed, commonly known as spread density. Figure 3a–c depicts the 2D and 3D reconstructed images of powders A, B, and C, respectively. Trapped gas was evident in powders A and B with a volume fraction of 0.7 and 0.2% respectively while no evidence of porosity was found in powder C. Trapped gas within the powder feedstock is an important quality parameter since it has been demonstrated that during AM processing, trapped gas can be released into the molten metal leading to rounded residual porosity in as-built parts [31]. 

The spread density obtained from powders A and B was consistent with the apparent density measurements obtained by the Hall, Carney, and Arnold meter methods with 54.7 and 53.6%, respectively. However, the apparent density obtained for powder C of 41.4% using micro-CT differs significantly from the traditional methods used.

### 3.4. Particle Segregation in A Powder Bed

**Figure 4:**
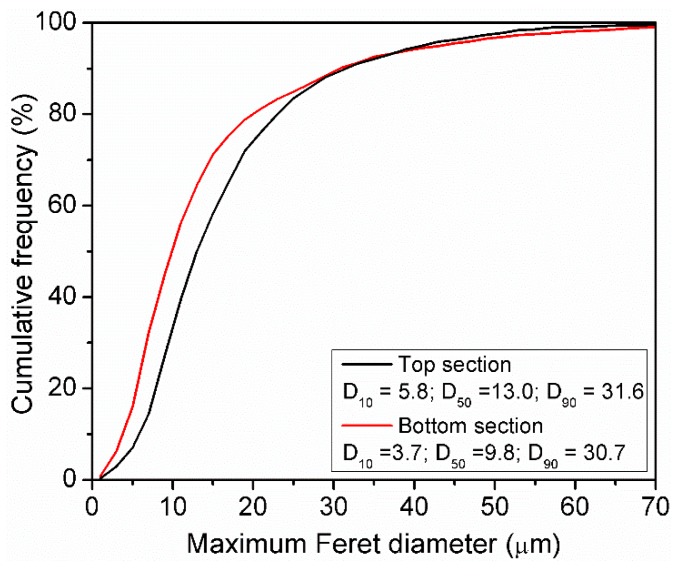
Particle segregation in a powder bed from the top and bottom sections of the building plate.

Little attention has been paid to particle segregation occurring during the powder recoating process. Differences in local PSD within a powder bed can have the potential to produce local variations of powder bed density during manufacturing and process instability in terms of melt pool signature [15]. In order to study the particle segregation during powder coating, a layer of 50 µm of powder C was spread over a previously laser rastered Al plate and the PSD was then analyzed at different locations over the plate. Figure 4, depicts the PSD of samples from powder C collected from the top and bottom sections of the Al plate. The top section corresponds to a position close to the beginning of the powder spreading while the bottom section corresponds to a position at the end of powder spreading. Differences in PSD between the top and bottom section of the powder bed were observed. Large particles with a D_50_ of 13.0 µm segregated in the region where spreading starts while smaller particles with a D_50_ of 9.8 µm, segregate at the end of the powder bed.

### 3.5. Water Vapor Adsorption Characteristics

**Figure 5:**
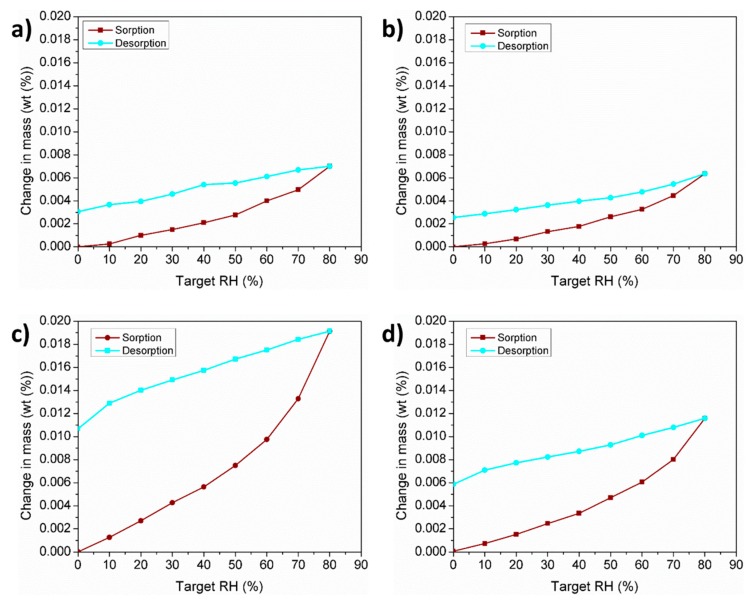
Dynamic vapor sorption (DVS) isotherms for (**a**) powder A, (**b**) powder B, (**c**) powder C, and (**d**) sieved powder C.

The water vapor adsorption test results of powders A, B, and C determined by means of DVS are shown in Figure 5a–c. In order to see the effect of PSD on the moisture adsorption, powder C was sieved to obtain a PSD of D_10_ = 48 µm, D_50_ = 65 µm, and D_90_ = 92 µm as to be comparable to powders A and B. The DVS isotherm for the sieved powder is presented in Figure 5d. Powder C has a significantly larger degree of vapor sorption compared to powders A and B. By eliminating the fine particles present in powder C, the degree of vapor sorption for the sieved powder C was reduced.

### 3.6. Multiple Cycle Flowability Test

**Figure 6:**
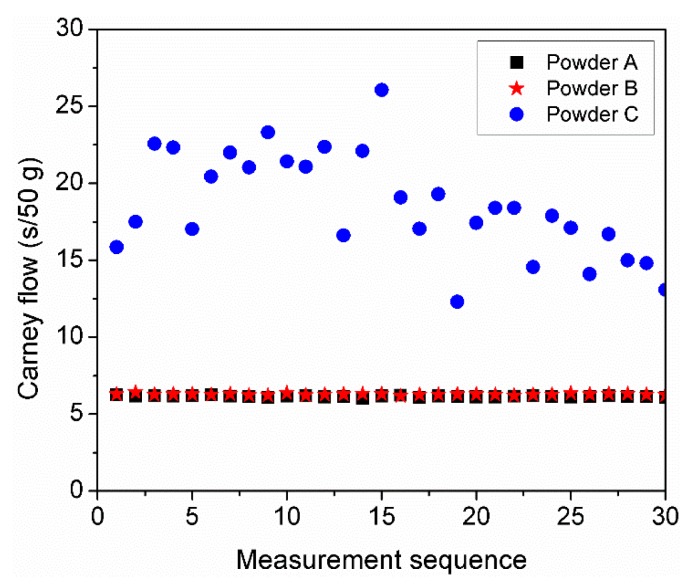
Carney flow variations of powders A, B, and C after oven drying and exposure to RH 40%.

In order to evaluate the effect of humidity on powder flow, a multiple cycle flowability test was carried out at a RH of 40% using 50 g of dried powders. Figure 6 shows the Carney flow corresponding to powders A, B, and C as a function of measurement sequence. No significant variation in flow was observed between powders A and B which remained stable over time with an average flow of 6 s. However, powder C showed a strong variation in flow within the first 5 min after removal from the furnace. During this time, the flow time increased from 16 s to approximately 22 s. The flow time then subsequently decreased continuously until reaching a flow time of 14 s after 30 consecutive measurements. The vapor sorption characteristics of the tested powders depicted by the previous DVS analysis (Figure 5) plays an important role in the powder flowability. It is well known that the surface of aluminum powders reacts with oxygen during powder production to form a passivation alumina layer. During powder handling, the passivated aluminum powders can absorb humidity and form a hydrated aluminum oxide [32,33]. The high surface area present in powder C due to the greater number of fine particles, increases the amount of adsorbed water which decreases the powder flow and/or contributes to the development of powder agglomerates [34]. After the first 5 min, it is believed that the observed increment in flowability may be related to the breaking up of the agglomerates due to the shearing strength produced by continuously flowing the same powder.

### 3.7. Particle Cohesiveness

**Figure 7:**
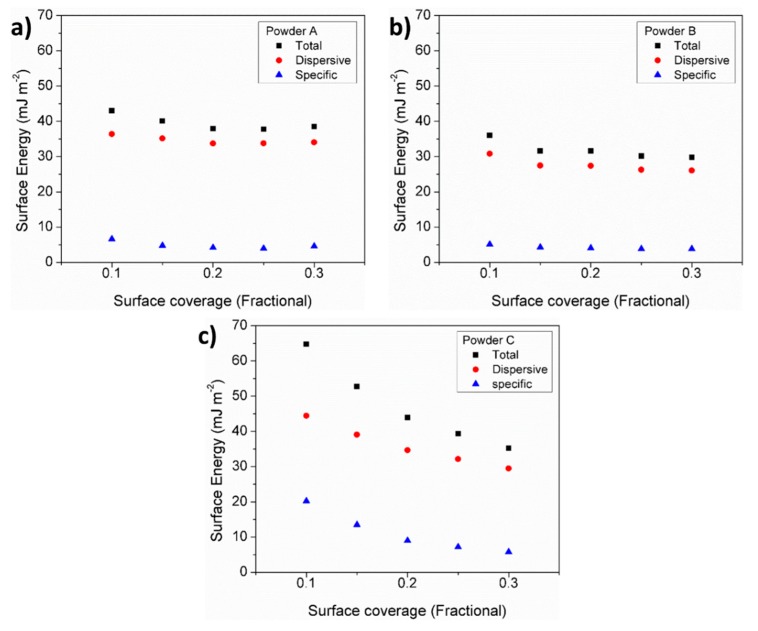
Surface energy of (**a**) powder A, (**b**) powder B, and (**c**) powder C.

Figure 7a–c, shows the surface energy as a function of surface coverage for powders A, B, and C, respectively. For all powders, the dispersive component of the surface energy was the most dominant. It can be seen that powder C displayed a much greater degree of surface energy heterogeneity, as indicated by the decrease in surface energy (dispersive and specific, leading to a decrease in total) with increasing surface coverage. The surface energy of powder C was also much greater than the other two powders at low surface coverage, which was likely to be due to the finer particle range present having a higher energy. Powders A and B have a higher degree of surface energy homogeneity, with the general trend of powder A having a greater surface energy across the entire surface.

**Figure 8:**
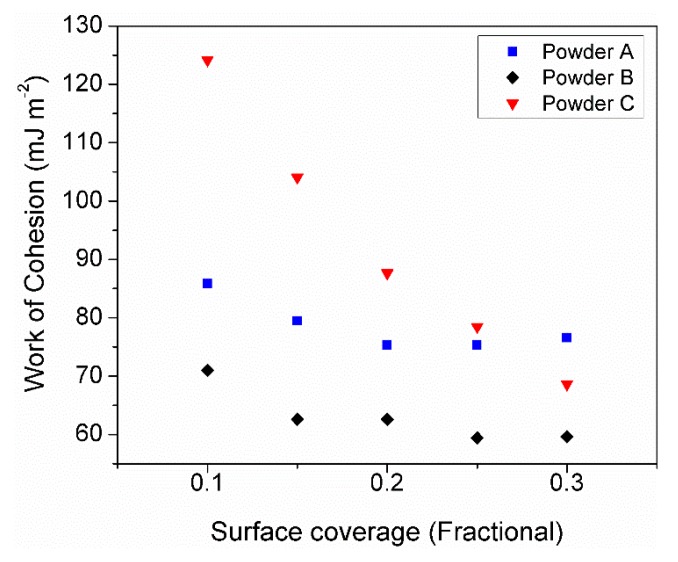
Comparison between the work of cohesion between powders A, B, and C.

The work of cohesion gives an indication of the natural affinity of the powders for agglomeration. Figure 8 shows the Work of Cohesion (Wco) of the powders A, B, and C. It can be seen that at the lowest surface coverage, powder C had a much greater Wco, which is most likely dominated by the fine particles present. Thus suggest that the presence of fine powders favors cohesion giving rise to powder agglomeration [34]. From this result, it would indicate that powder C would have the lowest flowability. 

### 3.8. Static and Dynamic Angle of Repose

During AM powder layer spreading, the powder is typically deposited in two steps: (1) the powder is piled up by gravity in front of a re-coating blade, and (2) the powder is spread over the powder bed by the horizontal movement of the re-coater. The first recoating step, may be properly represented by static flow tests while the second step, which is subjected to shear stresses due to the horizontal movement of the re-coater, might be better represented by studying the dynamic angle of repose. The static flow behavior for each powder characterized by the conical angle of repose is summarized in Table 4. It can be seen that the powder containing spherical and coarser particles, powder B, presents the lowest static angle of repose followed by powder A. In contrast, powder C which contains the largest amount of fine and irregular particles showed the highest static angle of repose.

**Figure 9:**
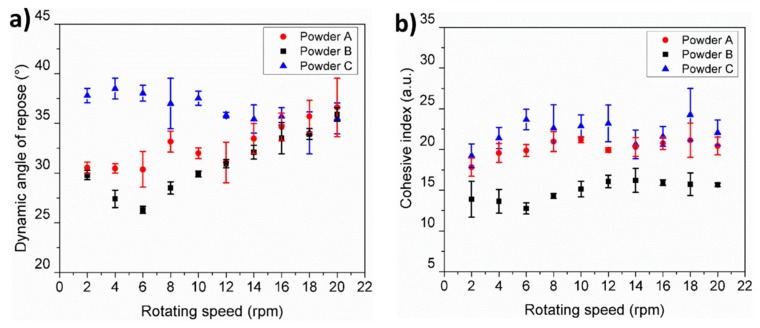
(**a**) Dynamic angle of repose and (**b**) cohesive index determined for powders A, B, and C.

Figure 9a presents the evolution of the dynamic angle of repose as a function of the rotating speed for powders A, B, and C. Dynamic powder flow measurements clearly show reproducible differences between the tested powders up to a rotating speed of 14 rpm. Below this speed, the spherical and coarser powders (A and B) show the lowest dynamic angles while the fine and non-spherical powders (C) present the highest angles of repose. At rotating speeds higher than 14 rpm, no statistical difference was observed. Specifically, powders A and B present a typical shear thinning behavior due to the slipping layers passing each other at rotating speeds between 2 and 6 rpm with a minimum dynamic angle of 26 ° and 30 °, respectively. At rotating speeds higher than 6 rpm, there was a transition from shear thinning to shear thickening behavior in both powders associated to the breakdown of layers and the formation of large aggregates [35]. The maximum angle of repose was obtained at 20 rpm for powders A and B with values of 37° and 36°, respectively. In contrast, powder C presents the highest dynamic angle of repose at the lower rotating speeds, i.e., between 2 and 6 rpm, with a maximum value of 38°. After 6 rpm, powder C shows a slight shear thinning behavior decreasing its angle of repose to an average minimum of 35° between 14 and 20 rpm. 

Quantification of the cohesion which occurs in the powders during drum rotation, can be carried out by studying the cohesive index which is determined from the fluctuations of the avalanche interface [29]. Higher values of cohesive index represent higher cohesion while a lower cohesion index represents lower cohesion between particles. Figure 9b depicts the evolution of the cohesive index as a function of the rotating speed for the tested powders. The coarser and spherical powder B, produced the lowest cohesion index whereas the finest and non-spherical powder C, presents the highest cohesion index. It was clearly seen that the particle cohesion of the Al powders was affected by the surface properties and PSD. The high surface energy and water adsorption characteristics measured in powder C compared with powders A and B, evidently increased the particle cohesiveness. Additionally, as the particles become smaller, the gravitational force contribution become negligible compared to the cohesive forces causing the powders to agglomerate and to reduce their flow [36,37].

## 4. Discussion

Ensuring process reliability and quality of additive manufactured components is one of the key challenges for widespread adoption of AM technologies. In LPBF, factors associated to laser optics, process parameters, and powder feedstock quality, have a direct effect on the process repeatability and quality of manufactured parts. Among these factors, powder feedstock quality is of paramount importance since changes in powder characteristics influence the laser-powder interactions resulting in process defects [38]. Baitimerov et al. [39], has shown the complex relationship between powder feedstock quality and printed part porosity. In their work powders with equivalent chemical compositions but different flow and apparent density characteristics, produced parts with relative densities between 94.4 ± 2.3% and 99.4 ± 0.3% using the same processing conditions. Thus, it has been demonstrated that flow and apparent density are important characteristics to consider due to the fact that homogeneous and highly packed powder layers are typically desired to enhance laser absorption [15].

Powder flow and apparent density are not independent properties, they are extrinsically related to other powder characteristics such as morphology, PSD, porosity, and surface chemistry. The particularities of powders A and B such as mono-sized PSD, high sphericity, good flowability and lack of satellite particles, produced a homogeneous powder bed with high apparent density similar to the one obtained by traditional powder characterization methods. However, the presence of large amounts of small and irregular particles such as the ones present in powder C, decreases the powder flow resulting in an inhomogeneous powder bed with low apparent density which is not comparable with traditional test results.

One of the main factors that influence the apparent density of a powder bed, is the presence of inter-particle forces which lead to cohesion [37]. It has been reported that non-cohesive and mostly spherical particles provide high apparent densities [40]. However, when cohesion between particles is present, agglomerates are likely to form giving rise to lower apparent densities [41,42]. The formation of agglomerates in the form of cage-like structures during packing have been observed by means of discrete element simulations when cohesive forces are taken into account. Such structures produce low apparent densities and are generated due to the presence of particles with high surface energy and cohesive forces typically larger than gravitational forces present [40]. 

The cohesiveness of the particles studied based on the measure of total surface energy, was evaluated by means of IGC. The total surface energy and work of cohesion measured in powders A, B, and C indicated that the PSD plays an important role in the total surface energy of these powders. Hence contributing to their cohesion behavior and consequently their flow. Powder C has a wide size distribution, with high surface energy at lower surface coverages, which was most likely due to the fine particles dominating. As the surface coverage increased, the lower energy sites of the fine particles adsorb the probe molecules, as do the larger particles in the system. It can be seen in Figure 7a that as the surface coverage increased the total surface energy of powder C tends towards that of the other two powders which contain the least quantity of fine particles.

The cohesiveness of the particles has also been determined using the rotating drum technique which provides a cohesion index based on the variation of the dynamic angle of repose. The results show that powder C presents the largest dynamic angle of repose and cohesive index followed by powder A and B. These results are in good agreement with the work of cohesion obtained by IGC. Additionally, this technique provides useful information regarding the flow of the powders at different shear rates. In the tested powders, shear thinning and shear thickening behaviors are observed depending on the rotation speed (shear rate). This observation provides insights into the critical powder spreading speed required to obtain the best flow characteristics for specific powders. This approach to obtain a value of ‘spreading speed’ cannot be obtained by traditional static flow measurements which only provide qualitative insight to the powder flow.

The presence of liquid bridges has been reported to increase the cohesion between particles due to the coalescence of the bridges leading to poor flow and low apparent densities [43]. In the present study, water adsorption tests have been carried out to investigate the water adsorption characteristics of powders A, B, and C. From the results, it was evident that a wide PSD with a large amount of fine particles such as powder C, led to higher amounts of adsorbed water. By sieving powder C to a PSD similar to that of powders A and B, the water adsorption behavior tends towards those of powders A and B. Fine particles would be more likely to adsorb a higher degree of water vapor relative to their mass due to the higher specific surface area. Multiple powder flow measurements carried out at constant RH of 40% indicate that humidity has an important effect on powder flowability depending on the PSD. Powder C, which adsorbed the highest amount of water, shows a significant change in flowability while powders A and B remained constant over time. 

Powder segregation occurring within the powder bed has a direct effect on the local apparent density. Discrete element method simulation results of the powder spreading process during AM processing [8,9], have shown evidence of particle segregation. Specifically, fine particles are segregated at the beginning of the spreading process while coarser particles are deposited at the end. In the present study, the cohesive powder C was tested to observe if particle segregation was likely to occur during the spreading process. In the present study, fine particles are segregated at the end of the powder bed while larger particles are present at the beginning. The simulation results provided in the literature contradict the ones observed experimentally within the present study. However, it is important to note that the discrete element simulations were carried out assuming free flowing spherical particles where the effect of surface energy and particle–particle interactions such as cohesion, were not considered. The flow behavior observed in non-cohesive and cohesive granular material differs significantly [44]. For the case of non-cohesive particles, axial segregation of larger particles is observed while in cohesive powders a lower degree of segregation is present [45]. Assuming that the shear strength during recoating between the coater and the particles is higher than the cohesion force of the agglomerates, the segregation observed in the present study could be explained by the breaking up of agglomerates during powder spreading resulting in the deposition of finer particles at the end of the powder bed.

## 5. Conclusions 

Traditional and non-traditional quantitative powder characterization techniques were used to evaluate the properties of different AlSi7Mg powders. The traditional characterization techniques are able to provide quantitative measurements of the powder performance in terms of flowability and apparent density. However, if the powders do not flow through the Hall and Carney funnels, no metrics can be obtained. In contrast to the traditional techniques, non-traditional techniques, such as CT scanning, dynamic vapor sorption, inverse gas chromatography, and the rotating drum technique, provide quantitative metrics of properties for free and non-free flowing powders. Additionally, they can be used to gain insight into characterisation parameters such as flow and apparent density. The results of this study have suggested that the presence of a PSD with a high number of fine particles facilitates water absorption and powder cohesion due to high surface energy. Such characteristics may hinder the spreading of uniform powder layers, which may lead to defects in AM parts. In contrast, when a narrow PSD and particles larger than 48 µm are present, water absorption and powder cohesion was decreased improving powder flow and apparent density.

## Figures and Tables

**Table 1:** Particle size distribution of the tested powders A, B, and C.

Powder	D_10_ (µm)	D_50_ (µm)	D_90_ (µm)	S_w_
Powder A	48	63	83	10.6
Powder B	54	70	91	10.8
Powder C	14	31	58	4.2

**Table 2:** Summarises the powder flowability of powders A, B, and C determined by Hall and Carney funnels.

Powder	Hall Flow (s/50 g)	Carney Flow (s/50 g)
Powder A	33.0 ± 0.4	6.1 ± 0.1
Powder B	32.7 ± 0.7	6.1 ± 0.1
Powder C	No flow	15.3 ± 0.4

**Table 3:** Apparent density of powders A, B, and C estimated by the Hall and Carney funnels as well as the Arnold meter.

Powder	Hall Apparent Density (%)	Carney Apparent Density (%)	Arnold Apparent Density (%)
Powder A	54.8 ± 0.5	55.5 ± 0.1	56.3 ± 0.1
Powder B	53.3 ± 0.5	54.8 ± 0.1	55.8 ± 0.1
Powder C	-	51.6 ± 0.4	52.7 ± 0.2

**Table 4:** Static angle of repose of particles A, B, and C.

Powder	Static Angle of Repose (°)
Powder A	30 ± 4
Powder B	26 ± 3
Powder C	39 ± 4
